# Simplifying the problem: metal salts can be active and controlled catalysts in polyester synthesis

**DOI:** 10.1039/d5sc05560a

**Published:** 2025-12-15

**Authors:** Mary Dana Czarinah L. Cheng-Tan, Zachary A. Wood, Megan E. Fieser

**Affiliations:** a Department of Chemistry, University of Southern California Los Angeles CA 90089 USA fieser@usc.edu; b Wrigley Institute for Environment and Sustainability, University of Southern California Los Angeles CA 90089 USA

## Abstract

Polymers (plastics) have become essential for daily life due to their versatility, providing low-cost solutions for transportation, food packaging, healthcare, and more. However, the growing accumulation of plastic waste highlights the urgent need for more sustainable approaches to polymer production. While discrete metal catalysts have shown control over certain polymer properties, their complexities can often limit broader use and commercial scalability. This Perspective explores simple metal catalysts for the synthesis of polyesters, a promising alternative for non-degradable commercial plastics for their chemical recycling potential. Simple metal salts may offer advantages such as reduced toxicity, cost-effectiveness, simplicity, and in turn, accessibility for more efficient and upscaled polymer screening. Recent discoveries show that simple metal salts can achieve high activity and control, suggesting they may be competitive with more complex catalysts. By focusing on catalyst simplicity, it could help bridge the gap between catalyst development and polymer design for a more holistic approach towards producing sustainable polymers.

## Abstract 2

Metal catalysts, with thoughtful consideration towards simplicity, can bridge the gap between commercial and designer catalysts in aliphatic polyester synthesis, offering examples of maintaining polymerization control without complicated synthesis.

## Graphical abstract

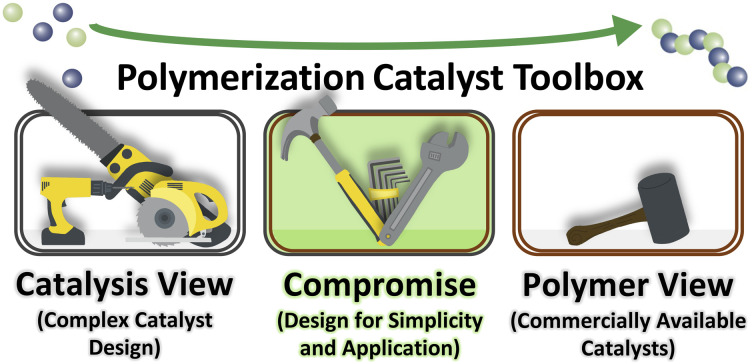

## Introduction

The commercialization of polymers for use in short- to long-term applications has transformed modern life by lowering the cost and/or energy needed for transportation, food preservation, comfort of living, and access to medical care.^1,2^ While polymers serve an important function when in use, their end-of-life has proven to be a persistent challenge that is only worsening.^3,4^ Solutions can come from many disciplines, such as engineering, chemistry, policy, design, or industrial infrastructure. However, if the goal is to make any substantial impact on reducing the amount of plastic pollution, major systemic changes must incorporate all solutions holistically.^5^

One key solution is the development of sustainable replacements for non-degradable polymers across as many applications as possible. While the market continuously provides access for long-term products to replace disposable items, such as metal straws, cotton tote bags, and reusable water bottles, these items are not often used enough to offset their economic and environmental costs of production.^6^ The end-of-life of these products is rarely better than the polymer products they were meant to replace. Replacement of polymer products with other materials, such as paper, aluminum, and glass, for short-term applications can be useful but has its own pitfalls.^7^ Another solution strives to recycle commercial polymer products for the same application or upcycle them into a higher-value material.^8–12^ While this strategy is valuable and necessary, it will not address all polymer waste produced. This can be due to the inability to reuse recycled polymer for the same application, the inefficiency in recycling/upcycling, and/or the limited demand for recycled polymers at the industrial scale. Finally, a reduction in the use of polymer products can be an important option, yet it relies on the general public's willingness to make lifestyle changes.

Another option chemists have been working on for decades is the synthesis of new polymers derived from more sustainable monomer sources (such as from biorenewable feedstocks) and end-of-life through recycling and composting.^12–14^ In addition to the beginning and end-of-life of these new materials, the polymers need to have the properties required for the target application to replace a non-degradable option. Once useful properties are identified for a sustainable polymer alternative, the scalable synthesis of these materials at competitive costs is one of the biggest challenges to direct commercial viability.^14,15^

Catalyst design has led to extraordinary activity and selectivity in the synthesis of these new polymers, yet many of these systems are not commercially viable. Designer catalysts are not often used outside of the particular research group that developed them. While there is no doubt that catalyst design is important to develop polymers with excellent properties, this Perspective argues that there is tremendous potential in the design of metal polymerization catalysts with extreme simplicity in mind. With a keen focus on simplicity while supporting the best polymerization activity, selectivity, and control, these systems could be more accessible for commercialization and broad use by polymer scientists. It is important to note that organocatalysts for polymerization are another emerging strategy, but these species rarely outperform metal catalysis in terms of polymerization rate and control and will not be discussed in this Perspective.^16–27^

Recent discoveries of simple and active metal catalysts for the synthesis of polyesters show promise for industrial use. Without a supporting ligand framework, simple metal salts can avoid increased toxicity concerns that complex ligand-based catalysts face.^28,29^ We define simple metal catalysts as commercially available metal salts, often in combination with co-catalysts that are also commercially available, which do not contain synthesized ligands that are not easily obtained commercially. By synthesizing complexes without complicated ligands, catalyst and polymer development can be more streamlined while also enhancing potential sustainability and commercial feasibility. Herein, the benefits and limitations of simple catalyst systems and opportunities for future discoveries will be discussed.

## Different perspectives between polymer and catalysis experts

**Fig. 1:**
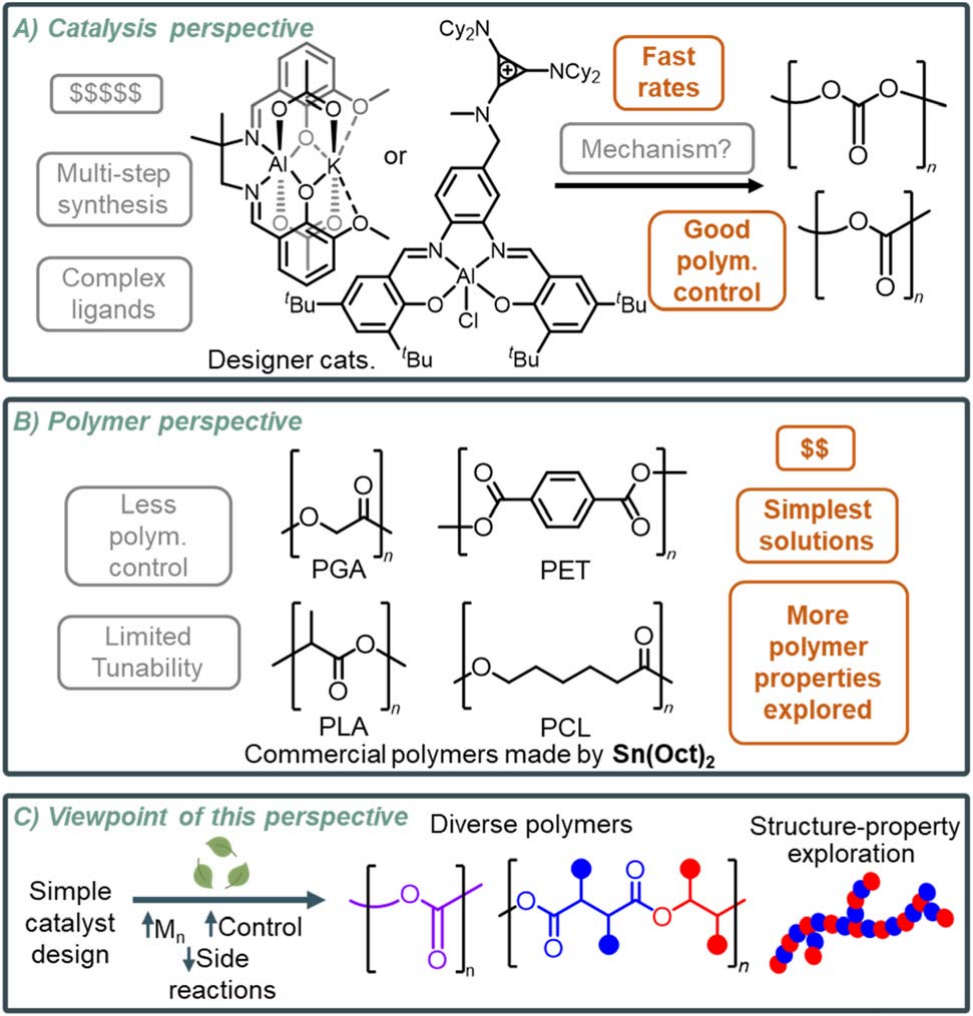
Overview of different perspectives both (A) catalysis and (B) polymer experts prioritize, and (C) this Perspective's viewpoint. Orange boxes indicate advantages of the respective field's perspective, and the gray boxes indicate the disadvantages of respective field's perspective.

Polymer and catalysis experts have long strived to develop sustainable polymers that are functional, scalable, and have a better end-of-life than current commercial polymers. However, the foci of the polymer field compared to the catalysis field do not always align (Fig. 1). Catalysis experts tend to delve into optimizing catalytic efficiency, selectivity, and polymerization mechanisms, emphasizing chemical interactions of the polymerization process more than scalability (Fig. 1A). On the other hand, polymer experts primarily explore the design, structure, and properties of the polymers, often driven by practical applications, such as packaging and healthcare (Fig. 1B). Rather than prioritizing the use of designer catalysts or even new catalysts, polymer experts tend to stick with catalyst systems they know will work well enough for large-scale synthesis, even if they are not the best in efficiency or for the environment. For instance, numerous groups focusing on catalysis have designed special ligands for poly(lactic acid) (PLA) to enhance efficiency and tacticity control of racemic lactide, while polymer experts may opt for a more direct and practical approach, commonly using l-lactide to avoid racemic mixtures and employing a commercial catalyst, such as tin octanoate (Sn(Oct)_2_).^30–35^ This catalyst is inexpensive, readily available, and durable under manufacturing conditions, which makes it desirable for larger-scale productions.

Despite these differences in their approach, we want to highlight that both fields bring great value in the pursuit of sustainable polymers. This Perspective argues that these two fields can meet in the middle to develop scalable catalytic strategies for polymer synthesis. Bridging both fields through collaborative interdisciplinary efforts can advance research for developing optimized, simple catalytic systems that can synthesize polymers with targeted physical and thermal properties while reducing environmental impact, overall offering a more comprehensive approach to diverse polymer synthesis (Fig. 1C). Moreover, interdisciplinary efforts can also encourage a circular economy by designing polymers that are both high-performing and able to participate in closed-loop recycling, whether it be depolymerization back to monomers or degradation into benign products. To achieve this, finding the right catalyst system that can accommodate polymer chemists is crucial. The development of catalyst systems that can balance selectivity, control, and reactivity with simple synthetic routes can further facilitate advances in sustainable polymer synthesis for commercial use.

This direction towards polymer synthesis is exciting as it can show the potential benefits of integrating multiple fields. While the development of designer catalysts is important, notable progress has been made toward catalyst efficiency and control for polymerization, particularly for tacticity control, it does not need to be the sole focus. There is significant potential for advances in the simplicity space without the need for complex ligands to achieve high enough activity, selectivity, and control of some emerging polymers. Catalyst designs that emphasize simplicity and usability without sacrificing performance are more likely to be used by polymer chemists, enabling a broader range of polymers. This approach, as indicated on Fig. 1C, can advance the sustainability aspect of polymer synthesis and application, contributing to the development of more environmentally friendly materials. However, prioritizing simplicity in catalyst design can make it challenging to maintain polymerization selectivity and control. This challenge makes one question what is sufficient selectivity and control for a polymer to have commercial potential with a cost-effective synthesis. This Perspective highlights recent advances in simple catalyst systems for the synthesis of sustainable polymers, showing their versatility and the opportunities they can present for future applications.

## Routes to polyesters

Carbonyl-containing polymers, including polyesters, polyamides, and polycarbonates, represent a class of materials that have increased opportunities for bio-based monomers and for polymers with achievable degradation/depolymerization routes. However, only a few carbonyl-containing polymers are made on a large industrial scale (Fig. 1B). To consider simplicity in catalyst systems for the synthesis of sustainable polymer alternatives, it is important to establish current commercial strategies to make similar polymers.

Poly(ethylene terephthalate) (PET) is the most widely commercialized polyester used today for packaging and clothing, among other applications.^36–38^ PET's recyclability, low cost, and versatility are what make this polymer valuable as a mass-produced polymer. As a step-growth condensation polymer, this polyester has been made with or without catalysts (often antimony trioxide). These methods are already commercially scalable, with targeted routes for mechanical and chemical recycling, and therefore not a key target for this Perspective.

In contrast, aliphatic polyesters such as PLA, poly(glycolic acid) (PGA), and poly(caprolactone) (PCL) are an important class of degradable polymers and have high potential for recyclability and biodegradability that can serve as alternatives to current non-degradable commercial plastics. These polymers are typically synthesized through ring-opening polymerization (ROP) of cyclic esters and are widely used in applications such as medical devices and agricultural films. While many groups have made catalyst systems that are highly selective and controlled with improved catalytic efficiency, commercial processes often continue to use a simple salt catalyst such as Sn(Oct)_2_, to produce these polymers due to its low cost yet high efficiency in making controlled polymers, and simplicity to synthesize.^39–41^ Even as catalysts become more intricate, implementing designer catalysts for industrial use is hindered by synthetic complexity and production costs.

While it is beneficial to have these commercial ROP polyesters, as they can give narrow dispersities and molar mass control in mild conditions, they are limited by their lack of structural diversity. To counter these limitations, ranging from thermal to mechanical properties, the polymers are sometimes copolymerized with another monomer or polymer to improve certain properties.^42–45^ This strategy is still limited in the properties it can achieve, requiring the need for more options.

Another route to making more property-tunable aliphatic polyesters is through the process of ring-opening copolymerization (ROCOP) of epoxides and cyclic anhydrides.^46–52^ With over 400 cyclic anhydride and epoxide pair combinations, many of which can be bio-derived, ROCOP can provide more versatile polyesters with targeted properties.^53^ This allows for a wide range of polyesters with tunable thermal, physical, and degradable properties that can be further adjusted *via* post-polymerization modification that can alter the esters on the backbone.

There are more polyester and other carbonyl-containing polymer structures in development, many of which come from bio-based resources and/or have a more sustainable end-of-life. However, the cost of synthesizing the polymers is often a major barrier to consider with regard to the commercial relevance of these materials, particularly as replacements for inexpensive polyolefins. This cost can even limit the scalable synthesis of new polymers in a research setting, preventing the understanding of the commercial potential of emerging materials. The economic feasibility of transitioning to sustainable polymers is dependent on the simplicity, efficiency, and cost-effectiveness of catalytic systems. By developing efficient and simple catalysts, a range of polymers can be accessed to facilitate the adoption of sustainable alternatives.

It is important to note that although there is a growing structural diversity of polyesters and other carbonyl-containing polymers, the more reactive nature of the carbonyl-containing functional groups of these polymers will limit the products they can be used for. However, this Perspective argues that the development of simpler, more cost-effective synthetic means for these polymers gives them the best potential to replace many applications that currently use polyolefins.

## Key concepts for developing new catalysts

Since the development and comparison of catalysts is the topic of interest for this Perspective, it is important to mention that proper benchmarks are needed in this field. However, there are more variables than commonly considered beyond the rate and selectivity of the desired reaction (avoiding undesirable side reactions). In the case of polymer synthesis, tacticity, regioregularity, control over molecular weight growth (living polymerization), dispersity control, ability to acquire high molar mass polymers, and catalyst stability in the presence of chain transfer agents (immortal polymerization) should also be considered when relevant. Some of these variables will be more important for the commercial potential of a material. For example, the ability to synthesize high molar mass polymers is expected to be more important than dispersity control.

When comparing two catalysts, different conditions may be necessary to optimize these variables. In some cases, comparing two catalysts at identical conditions can allow for a specific comparison, while other cases may discuss comparisons of different catalysts at their selected optimized conditions. When possible, to allow for direct comparisons to other catalysts in the literature, one can run the reactions at the same optimized conditions as the catalyst in the literature. However, it is important to note that the new catalyst may not react best under those conditions. Due to the complicated synthesis of designer catalysts, it can be a burden to resynthesize a literature catalyst to compare to your system under truly identical conditions. This gives further support to the use of the simple salts advised in this Perspective, as comparison to another catalyst system should be a much more reasonable task.

Nonetheless, when arguing a specific behavior of a catalyst, repeatability is crucial. Therefore, reactions done more than once will give larger credibility to the finding. Control reactions should also be done to identify the importance of different variables in a polymerization reaction.

## Simple salts for controlled polymerization

This Perspective encourages the focus on simplicity in catalyst design for new polyesters to be competitive with current commercial plastic production. An ideal catalytic method would be inexpensive, fast, and selective (avoiding undesirable side reactions), and would be able to show controlled molar mass growth, reasonable dispersity control, and stability to chain transfer agents. Because of this range of variables, it can be difficult to benchmark specific traits that make a catalyst system “the best”, as it requires a combination of these variables.

**Fig. 2:**
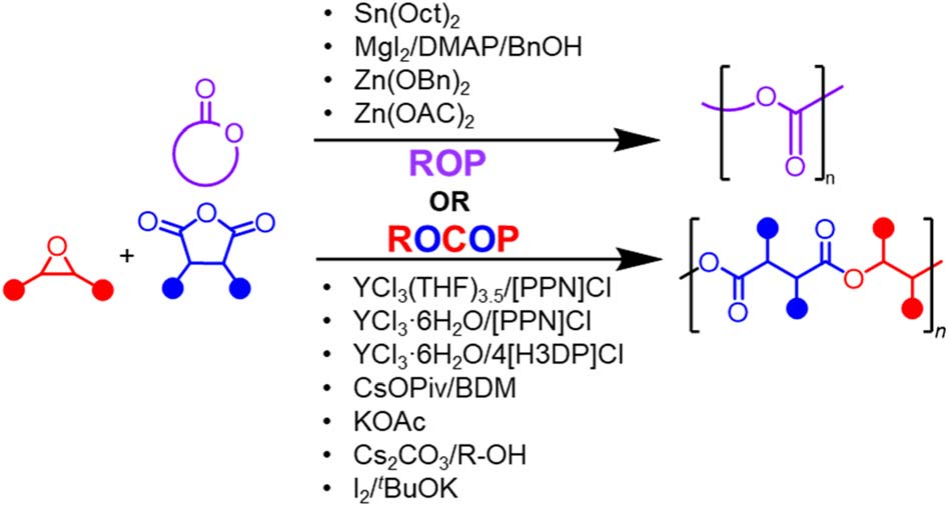
Examples of simple salts used for polyester synthesis.

Monomers can be costly, especially ones that are bio-sourced, so making the catalyst inexpensive and recyclable is an important step in reducing overall production costs. Several studies have demonstrated that readily available metal salts, such as magnesium-,^54,55^ potassium-,^55–59^ zinc-,^55^ cesium-,^58,60–67^ yttrium-,^68–70^ and lanthanide-based catalysts^70,71^ (Fig. 2) can effectively produce aliphatic polyesters with minimal side reactions, narrow dispersities, and controlled molar masses.

**Fig. 3:**
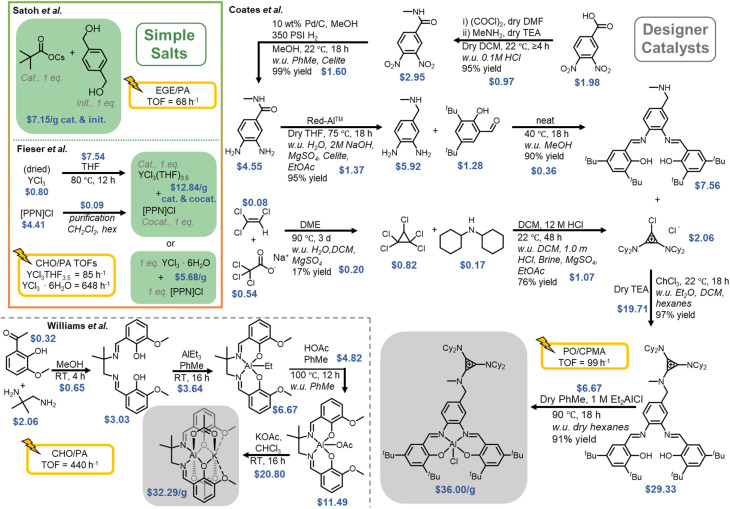
Cost *vs.* performance overview of catalysts used for polyester synthesis. w.u. is the work-up to purify/filter/dry the crude products. Prices for chemicals have been obtained from vendors in the article's SI if listed. For general chemicals, pricing was obtained consistently from a single vendor for each compound throughout the calculations. Specific monomer pairs were chosen to represent the TOFs for a better comparison between different catalyst systems.

Many studies have primarily focused on complex catalyst systems for polyester synthesis, particularly for ROP or ROCOP. These complexes can often involve high costs, limited scalability, and multi-step processes (Fig. 3).^46–52^ While these systems have demonstrated high efficiency and selectivity, emerging simple catalytic approaches are highly competitive in terms of selectivity, control, and efficiency. These catalysts can advance polymer sustainability approaches, as many are commercially available or simple to synthesize, thus reducing time and costs in commercial production. Fig. 3 shows a comparison of the cost of synthesis for two of the best designer catalysts to date, developed by the Coates^46,72^ and Williams^47,73^ groups, in comparison to simple catalyst systems developed by the Satoh^60^ and Fieser^68^ groups for the ROCOP of epoxides and cyclic anhydrides. When accounting for the energy cost, time involved, and waste produced in the synthesis of designer catalysts, which would require life cycle assessment (LCA) and technoeconomic analysis (TEA), the economic advantage of simple salt catalyst systems is notable, as these expenses accumulate. Even without accounting for the additional costs, simple salt systems are less than half the cost of the designer analogues. While on a gram scale, a difference of ∼$20 may seem small, on an industrial scale, these costs add up substantially and will be an obvious choice for large-scale production of polymers. When scaled up, the added expenses of energy cost, time, and waste handling further widen the cost gap. These comparisons identify the value of simple systems if they can maintain the desired selectivity and control of polymerization. Considerations should be made to ensure simple salt solutions are more cost-effective than designer catalysts and perhaps less harmful to the environment. In some cases, a designer ligand with an abundant metal may be more attractive than an expensive, less abundant metal salt.

Examples for the ring opening polymerization of cyclic esters have limited simple metal catalyst examples, yet some exciting findings have emerged. Dove and coworkers found that combining Lewis bases (such as N-heterocyclic carbene (NHC), 1,8-diazabicycloundec-7-ene (DBU), and 4-dimethylaminopyridine (DMAP)) with simple Lewis acids, like MgX_2_ in the ROP of lactones led to catalytic activity that followed periodic trends in which larger, less electronegative nucleophiles (X = I > Br > Cl) had the highest activities.^54^ Garden and coworkers have also reported on a range of divalent metal alkoxides (using Mg, Ca, Zn) for the ROP of lactide, identifying Zn(OBn)_2_ (Bn = benzyl) as the most active catalyst.^55^ This system produced PLA with high *M*_n_ (up to 157.5 kg mol^−1^) and good dispersity control (*Đ* = 1.14) using high monomer loadings. Additionally, while still requiring a few steps to synthesize, Garden and coworkers also reported on a potassium bifunctional salt that was able to effectively produce a perfectly alternating epoxide and cyclic anhydride sequence, subsequently producing a block copolymer with vinyl acetate.^56^

Alkali metal carboxylates and rare earth metal halides are the two main classes of simple salts that have emerged as catalysts for the ROCOP of epoxides and cyclic anhydrides. Li and coworkers found that commercially available and food-safe alkali metal carboxylates, with potassium acetate (KOAc) being the most active, are effective catalysts for the ROCOP of epoxides and cyclic anhydrides.^57^ Xu and coworkers expanded this approach by investigating readily available Na, K, and Cs carbonates for the ROCOP of phthalic anhydride (PA) and epoxides, finding that Cs_2_CO_3_ in combination with an alcohol initiator showed the highest catalytic activity while minimizing side reactions.^58^

Further contributions from Satoh and coworkers demonstrated that several alkali metal carboxylates with 1,4-benzenedimethanol (BDM) as an initiator are active for the ROP of a range of epoxides,^63,64^ cyclic esters,^61,62,65,66^ and ROCOP of both epoxides and cyclic anhydrides^60–62,64,65^ for different chain sequences or topologies (such as the monomer mixtures PA/ethyl glycidyl ether (EGE) for perfectly-alternating monomers in a linear chain,^62^ PA/carbic anhydride (CPMA)/EGE for block copolymers,^61^ and PA^COOH^/1-butene oxide (BO) for hyperbranched polymers^60^). It was observed that catalytic activity increased with cation size and electron-donating substituents on the alkyl group, with cesium pivalate (CsOPiv) being the most active catalyst. Chakraborty and coworkers further developed a cooperative dual catalytic system by introducing an equivalent amount of molecular iodine with various alkali metal alkoxides, with I_2_/^*t*^BuOK showing the best activity.^59^

The Fieser group has developed catalyst systems capable of handling diverse monomer mixtures and investigated how the catalysts can handle these monomer mixtures. Notably, it was found that YCl_3_THF_3.5_ and YCl_3_·6H_2_O, with bis(triphenylphosphoranylidene)ammonium chloride ([PPN]Cl) as a cocatalyst, were highly efficient for the ROCOP of epoxides and cyclic anhydrides with minimal side reactions, having the highest turnover frequency for BO-*alt*-CPMA polymer at 402 h^−1^ using the anhydrous catalyst.^68^ This has been extended to incorporate monomer mixtures that allow for tailored gradient, random, and block architectures.^69^ In comparison to Cs salts reported by Satoh,^60–66^ these yttrium salts showed more options for gradient copolymers with a larger tapered region. Extensions to the lanthanide series show a more subtle tuning in the copolymerization of epoxides with two anhydrides than when shifting from Y to Cs.^71^

Additionally, it was found that rare-earth metal ionic liquids (MILs), synthesized with a 1 : 4 ratio of metal chloride salt-to-phosphonium ionic liquid, were effective for ROCOP.^70^ These MILs facilitated the polymerization with minimal side reactions and achieved some of the highest reported molar masses for specific monomer pairs, in which the exposure of the MIL to water or air greatly affected the activity of the catalyst. By introducing simpler, yet effective catalyst systems with competitive outcomes, this offers a more approachable route for translating these catalyst systems to more industrial applications.

The use of simple metal catalyst systems has significant potential for polymerization, however, there are limitations that must also be considered, especially with tacticity control, which can affect a polymer's thermal, mechanical, and degradation properties. While multiple factors can impact tacticity control, ligands play an important role wherein a chiral coordination environment is often necessary for stereoselective placement.^74,75^ Designer catalysts can offer fast rates and great control, but they can come with challenges such as limited stability or complex synthesis. This comes with a trade-off: though the basic metal catalysts offer a more simplistic synthesis and a higher potential for scalability, these catalysts may exhibit slower polymerization rates or reduced selectivity. Therefore, it is essential to determine when a simple catalyst system is sufficient or whether a more complex catalyst system is needed. While not directly covered in this Perspective, the ideas presented herein are expected to apply to the synthesis of other carbonyl-containing polymers, including polyamides,^76,77^ synthesized from ROP of cyclic amides, and polycarbonates,^78–81^ synthesized through the ROP of cyclic carbonates or ROCOP of epoxides and carbon dioxide.

## High molar mass polymers

Achieving high molar mass is especially important for polymers as molar mass significantly influence a polymer's mechanical and thermal properties.^82,83^ To better understand the structure–property relationship of polymers, high molar mass polymers are required. As the long, flexible polymer chains become entangled, the more restricted the mobility of the chains becomes, giving the polymers certain properties like strength and viscosity. In order to understand the entanglement molar mass (*M*_e_) of an emerging polyester, it will be necessary to develop catalytic methods to controllably obtain a range of high molar masses at a reasonable scale. For polyesters from the ROCOP of epoxides and cyclic anhydrides, only one study has been conducted to acquire the entanglement molar mass for polyesters with varied epoxides and phthalic anhydride.^84^ In this case, the *M*_e_ ranged from 13 to 50 kg mol^−1^ across five epoxides, suggesting the range can be very large as the structure varies. This validates the need to make a range of molar mass polyesters with different structures to determine how high is “high enough” molar mass for commercial application.

Others have used more intricate catalysts to achieve high molar mass polymers.^47,52,72,85–87^ However, highly reactive designer catalysts can often show chain termination before high molar mass polymers can be achieved. Simple catalyst systems have also achieved comparable or higher molar mass products. Satoh and coworkers have previously achieved high molar mass polyesters *via* the ROCOP of epoxide and cyclic anhydrides using CsOPiv as a catalyst without the need for a cocatalyst.^60–66^ In order to achieve polymers with molar masses as high as 27.7 kg mol^−1^,^64^ an extreme excess of monomers was required with long reaction times.

The Fieser group demonstrated that the YCl_3_THF_3.5_ catalyst and [PPN]Cl cocatalyst combination was able to attain molar mass twice the theoretical molar mass expected for a BO-*alt*-CPMA polymer (as high as 302.2 kg mol^−1^) with a narrow dispersity and minimal side reactions. This increase in molar mass is attributed to coupling two chain ends past their full anhydride conversion, a polymer termination reaction unique to this simple salt catalyst system.^68^ Further, the Fieser group has seen an even faster way to achieve high molar mass ROCOP polymers with BO-*alt*-CPMA using a yttrium-based MIL as the catalyst, which showed moderate dispersity control and minimal side reactions.^70^ While the rate of obtaining high molar mass polymers is always much higher (as high as 185 kg mol^−1^ h^−1^) than other catalysts in the literature (<10 kg mol^−1^ h^−1^), the obtained molar mass of the polymers was found to be inconsistent across experiments. This inconsistency is presumably due to inconsistent initiation efficiency for these catalysts, indicating an area for improvement. These findings suggest that simple metal salts can provide opportunities to achieve controlled high molar mass polymers with moderate dispersity and minimal side reactions.

## Complex topologies

**Fig. 4:**
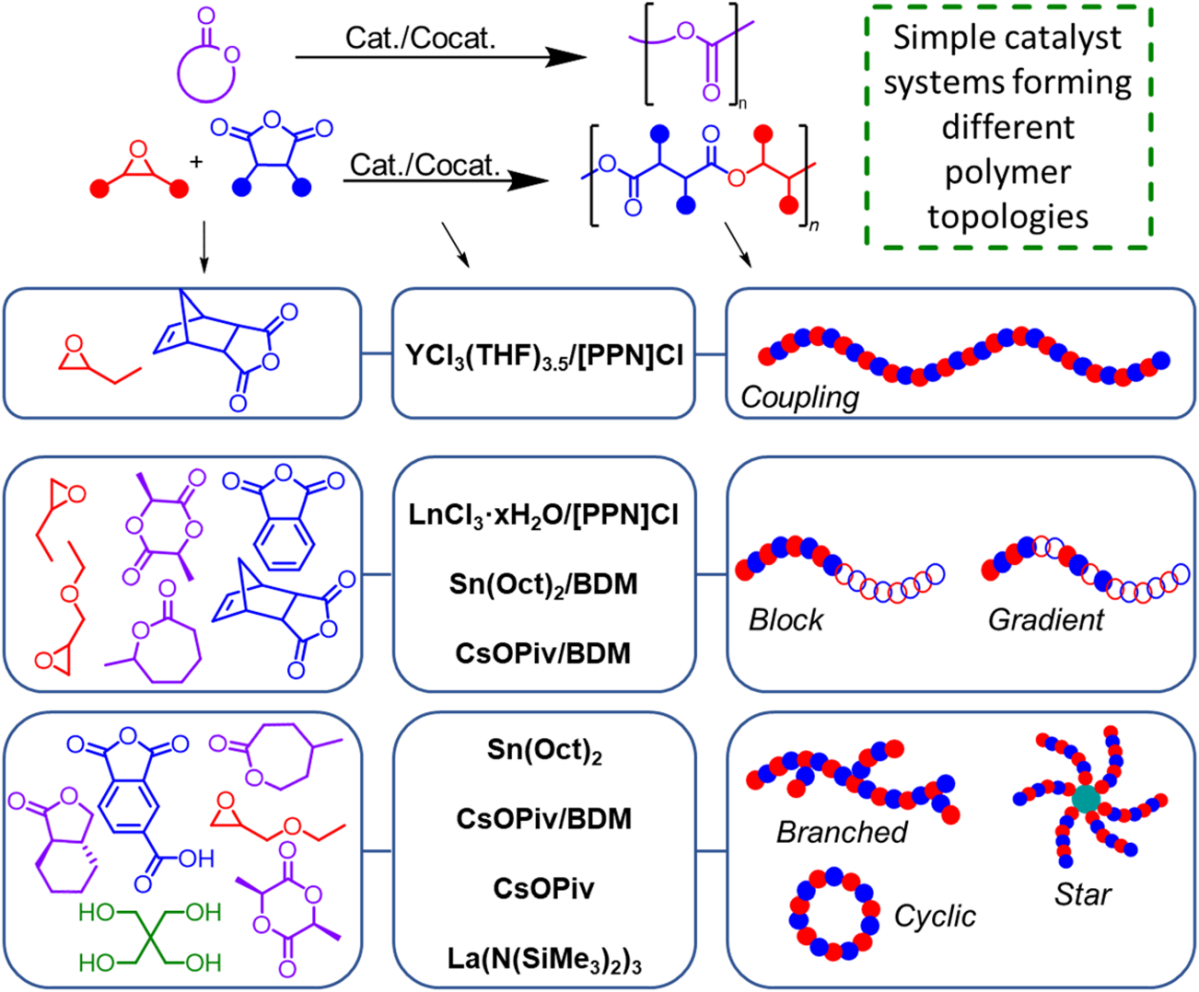
Examples of simple salts for the polymerization of polyesters with different topologies.

Adjusting the polymer topology can be another way to adjust the thermal and physical properties of polymers, such as the polyesters discussed in this Perspective. As simple salt catalysts have shown potential for producing polymers with good molecular weight and dispersity control at fast rates, they have also enabled the synthesis of a variety of copolymer classes such as block, gradient, graft, branched, and star copolymers (Fig. 4). Many aliphatic polyesters have been synthesized commercially and studied in the form of a linear topology through ROP, such as PLA, PCL, and PGA. Though still beneficial as linear polymers, these exhibit certain property limitations that can be improved by copolymerizing specific polymeric segments in different ways to tune and diversify polymer mechanical, thermal, and degradation properties.^88^ For example, to improve PLA's inherent brittleness, it can be copolymerized with ε-caprolactone, wherein the PLA's hard blocks are combined with PCL's soft blocks to increase polymer rigidity and flexibility.^89,90^ PLA can also be copolymerized with PGA to enhance degradation capability, or with polyethylene glycol (PEG) to improve polymer hydrophilicity in biomedical applications.^42–44^

Satoh and coworkers were able to achieve co-polyether,^63^ co-polyesters,^60–62,64–66^ and polyether–polyester^63^ block copolymers by using cesium and potassium carboxylates with a BDM initiator, using combinations of epoxides, cyclic anhydrides, and cyclic esters. In this case, block copolymers were almost always observed with complex monomer mixtures. The Fieser group has shown that by using a range of commercially available rare-earth metal hydrate salts LnCl_3_·6H_2_O (where Ln = La, Ce, Nd, Sm, Gd, Y, Ho, Tm) with [PPN]Cl, different copolymer sequences can be achieved while still maintaining polymerization control for mixtures of multiple epoxides and/or anhydrides.^69,71^ Depending on the monomer pairs and the Lewis acidity of the metal, more complex topologies like block, gradient, and random copolymers can be achieved. Hillmyer and coworkers have used Sn(Oct)_2_ to make a triblock copolymer 1,4-benzenedimethanol (BDM) as an initiator with 2- and 6-methyl-ε-caprolactone and l-lactide as the monomers, further finding that this triblock copolymer can be used for pressure-sensitive adhesives.^91–93^

Beyond conventional linear polymers, varying the structural configuration of a polymer with similar composition is known to distinctly exhibit different mechanical and thermal properties. Linear polymers can pack more efficiently into a crystal lattice as they can stack upon each other due to their structural regularity.^82^ This results in polymers that are denser with higher crystallinity, thermal resistance, and greater mechanical strength, which can be used in applications that require rigidity and strength, such as milk jugs and detergent bottles. On the other hand, branched polymers have an irregular structure that hinders the polymer from packing tightly, which then leads to lower crystallinity and density. These polymers can subsequently be used for applications that require more flexibility and durability, such as water bottles and shopping bags.^82,94,95^ One approach to produce ROCOP hyperbranched polyester involves a complex catalyst system with a monomer containing a functional group that serves as a chain transfer initiator.^72^ Satoh and coworkers used a CsOPiv and BDM catalyst system to copolymerize an anhydride functionalized with a chain-transfer carboxylic acid group with various epoxides to make hyperbranched polymers.^60^ By introducing l-lactide in the system, they produced a core–shell-type block copolymer that consists of a hyperbranched polyester core and a PLLA outer shell,^62^ along with another core–shell type block copolymer using other monomer combinations.^61^ Further exploration in control of branching density and the influence on polymer properties is needed.

Star polymers, a type of branched polymer, can be synthesized by selecting a multifunctional initiator in conjunction with a catalyst, forming multiple arms projecting from the core. Due to its unique globular structure and smaller hydrodynamic radius compared to linear polymers, it also has the potential to be used in biomedical applications, such as drug delivery and antimicrobial agents.^96^ Satoh and coworkers reported a three-armed star block polymer with an anhydride/epoxide polyester with poly(l-lactide) (PLLA) using a CsOPiv and trimethylolpropane^62^ or 1,3,5-benzenetrimethanol^65^ catalyst system. When switching the initiator to a pentaerythritol, they were able to obtain a four-armed star block polyester.^65^ Additionally, they investigated the ROP of an epoxide with CsOPiv and trimethylolpropane, resulting in a three-armed star polyether.^63^

Hillmyer and coworkers have also explored different topologies, such as graft copolymers for bottlebrush architecture, using a Grubbs generation III catalyst to carry out a ring-opening metathesis copolymerization. By copolymerizing a poly((±)-lactide) ω-norbornenyl macromonomer with a dimethyl-ester norbornene comonomer, they synthesized a random graft polymer with a poly(norbornene) backbone and PLA side chains. They have additionally reported synthesizing star and star–block copolymers with Sn(Oct)_2_ and a multi-functional alcohol initiator.^97–100^ Since the polymers are synthesized using commercially available catalyst systems, this allowed them to focus more on the structure–property relationships and their potential for applications without being limited by complex catalyst synthesis.

Cyclic polymers have increasingly gained attention due to their unique structural and physical properties. Unlike linear polymers, cyclic polymers lack chain ends, resulting in decreased chain mobility and increased thermal stability.^101,102^ Cyclic polymers' absence of chain ends allows them to have limited reptation, the snake-like motion of polymer chains. The polymer chains can only entangle in limited ways, contributing to unique physical properties that generally lead to lower solubility and intrinsic viscosities with increased crystallinity, glass transition temperature (*T*_g_), and melt temperature (*T*_m_), compared to linear analogues.^103,104^

Simple metal systems have shown to be effective catalysts for cyclic polyester synthesis. Bourissou and coworkers used Zn(C_6_F_5_)_2_ with an amine or phosphine for the ROP of lactide and ε-caprolactone, producing cyclic polyesters of lactide, caprolactone, or block copolymers of both.^105^ Weidner and coworkers identified that cyclic PLLA can also be synthesized by Sn(Oct)_2_ through a ring-opening polymerization with simultaneous polycondensation mechanism^106^ and a commercially available Bu_2_SnO by ring-expansion polymerization.^107^ Bourbigot and coworkers synthesized cyclic PLLA using a Mg(BH_4_), Ca(BH_4_)(THF)_2_ and Ln(BH_4_)(THF)_3_ (where Ln = La, Nd, Sm), finding that the lanthanide borohydride complexes showed the highest catalytic activity.^108^ In addition to bulk polymerizations of l-lactide, they used the lanthanide borohydride catalysts for polymerization *via* reactive extrusion, wherein the monomer and catalyst are continuously mixed and fed through an extruder, potentially making the process scalable for larger-scale productions. Chen and coworkers have successfully used a commercially available Ln[N(SiMe_3_)_2_]_3_ (Ln = La, Sm, Y) to isolate a cyclic polymer from the ROP of γ-butyrolactone and 4,5-*trans*-cyclohexyl-fused γ-butyrolactone at room temperature (Fig. 4).^109^ Instead of forming linear polymers upon chain termination, an intramolecular backbiting reaction occurs to obtain the cyclic polymer. There was a noted decrease in catalytic activity as the size of the lanthanide decreased, with lanthanum yielding the most efficient results. Notably, they found that the cyclic analogue has greater thermal stability than its linear analogue by using thermal gravimetric analysis (TGA). Additionally, while their *T*_g_ was comparable, the cyclic polymer displayed lower intrinsic viscosity, which is due to a smaller hydrodynamic volume.^109–111^ These findings motivate further investigation and understanding of the structure–property relationship of cyclic polymers compared to their linear analogues to understand potential applications with more tailored thermal and mechanical properties.

## Conclusion

The development and understanding of simple metal salt catalysts for practical application in polyester syntheses will benefit from the collaboration of catalysis and polymer scientists who bring complementary perspectives to the work. Identifying and optimizing factors that can improve the reactivity of simple metal salts, in terms of *M*_n_ and dispersity control with minimal side reactions, can be beneficial in expanding the scope of polymers and their structure–property relationships. Even without an intricate ligand design, simple metal salts can still be tuned to change their reactivity without complex synthesis, including their metal and cocatalyst identity, or using other commercially available or easily synthesized ligands. There are still many options to pursue, between different metal salts that could continue to show unexpected polymerization activity. As mentioned in this Perspective, other additives can be simple, yet provide improved activity for a salt, including salt cocatalyst, Lewis bases, and ionic liquids. Simple metal salts should be tested in areas that are typically dominated by complex catalyst systems.

Going forward, building effective collaboration and communication between catalysis and polymer groups is essential. It should be expected that innovations in catalysis should lead to use by polymer chemists. This opportunity for collaboration allows for many benefits. Catalysis experts can continue to tune systems to find the catalysts that are good enough, yet simple enough for use by polymer experts. Polymer experts can then explore the scalability and the monomer scope for these simple catalysts. This mutually-beneficial collaborative space can be achieved by having an understanding of shared goals and priorities, with a particular emphasis on the polymerization outcomes most needed for a particular polymer. The following is a list of recommendations that catalysis and polymer experts can implement to advance this idea.

For catalysis experts:

• Focus on simple catalyst systems that are inexpensive, fast, and selective with good polymerization control (produce low dispersity polymers with high molar masses).

• Develop a more standardized benchmarking, with an emphasis on what variables matter most for a particular polymer.

• Identify the levers that can be pulled to tune a simple system to improve a catalyst's activity without straying from methods that can be easily pursued by others.

For polymer experts:

• Consider using more commercially available catalysts that come from the catalysis groups, providing validation on the reproducibility of developed systems.

• As polymer properties are shown to be promising, communication should be made with catalyst groups to focus their efforts.

• Explore the scalability of new catalyst systems for bulk polymer synthesis that allows for polymer properties to be measured.

By emphasizing catalyst simplicity, it can help bridge the gap between catalyst development and polymer design, which can encourage a more integrated approach to sustainable polymer production. Perhaps, this strategy could allow for faster development of commercially viable polyesters to replace non-degradable materials, productively addressing polymer pollution.

## Author contributions

MDCLCT and ZAW wrote the manuscript. MEF conceptualized, edited and directed the writing of the manuscript.

## Conflicts of interest

There are no conflicts to declare.

## Data Availability

Citations are provided for all data discussed in this Perspective.
